# Derivation and validation of a clinical predictive model for longer duration diarrhea among pediatric patients in Kenya using machine learning algorithms

**DOI:** 10.1186/s12911-025-02855-6

**Published:** 2025-01-15

**Authors:** Billy Ogwel, Vincent H. Mzazi, Alex O. Awuor, Caleb Okonji, Raphael O. Anyango, Caren Oreso, John B. Ochieng, Stephen Munga, Dilruba Nasrin, Kirkby D. Tickell, Patricia B. Pavlinac, Karen L. Kotloff, Richard Omore

**Affiliations:** 1 Kenya Medical Research Institute- Center for Global Health Research (KEMRI-CGHR), P.O Box 1578-40100, Kisumu, Kenya; 2 Department of Information Systems, University of South Africa, Pretoria, South Africa; 3 Department of Medicine, Center for Vaccine Development and Global Health, University of Maryland School of Medicine, Baltimore, MD USA; 4 Department of Global Health, University of Washington, Seattle, USA

**Keywords:** Machine Learning, Longer duration diarrhea, Pediatric, Prediction

## Abstract

**Background:**

Despite the adverse health outcomes associated with longer duration diarrhea (LDD), there are currently no clinical decision tools for timely identification and better management of children with increased risk. This study utilizes machine learning (ML) to derive and validate a predictive model for LDD among children presenting with diarrhea to health facilities.

**Methods:**

LDD was defined as a diarrhea episode lasting ≥ 7 days. We used 7 ML algorithms to build prognostic models for the prediction of LDD among children < 5 years using de-identified data from Vaccine Impact on Diarrhea in Africa study (*N* = 1,482) in model development and data from Enterics for Global Health Shigella study (*N* = 682) in temporal validation of the champion model. Features included demographic, medical history and clinical examination data collected at enrolment in both studies. We conducted split-sampling and employed K-fold cross-validation with over-sampling technique in the model development. Moreover, critical predictors of LDD and their impact on prediction were obtained using an explainable model agnostic approach. The champion model was determined based on the area under the curve (AUC) metric. Model calibrations were assessed using Brier, Spiegelhalter’s *z*-test and its accompanying *p*-value.

**Results:**

There was a significant difference in prevalence of LDD between the development and temporal validation cohorts (478 [32.3%] vs 69 [10.1%]; *p* < 0.001). The following variables were associated with LDD in decreasing order: pre-enrolment diarrhea days (55.1%), modified Vesikari score(18.2%), age group (10.7%), vomit days (8.8%), respiratory rate (6.5%), vomiting (6.4%), vomit frequency (6.2%), rotavirus vaccination (6.1%), skin pinch (2.4%) and stool frequency (2.4%). While all models showed good prediction capability, the random forest model achieved the best performance (AUC [95% Confidence Interval]: 83.0 [78.6–87.5] and 71.0 [62.5–79.4]) on the development and temporal validation datasets, respectively. While the random forest model showed slight deviations from perfect calibration, these deviations were not statistically significant (Brier score = 0.17, Spiegelhalter *p*-value = 0.219).

**Conclusions:**

Our study suggests ML derived algorithms could be used to rapidly identify children at increased risk of LDD. Integrating ML derived models into clinical decision-making may allow clinicians to target these children with closer observation and enhanced management.

**Supplementary Information:**

The online version contains supplementary material available at 10.1186/s12911-025-02855-6.

## Background

Diarrhea is still a significant global public health problem causing approximately 1.7 billion episodes and 443,832 deaths annually among children < 5 years [1]. This burden is disproportionately high in low- and middle-income countries (LMICs). Longer duration diarrhea (LDD) defined as diarrhea episode lasting ≥ 7 days encompasses both prolonged acute diarrhea (7–13 days) and persistent diarrhea (≥ 14 days) [2]. Despite current diarrhea management guidelines, which focusses on acute diarrhea, up to 20% of diarrhea cases in LMICs end up becoming LDD [3, 4]. Whilst LDD represents a relatively small portion of childhood diarrheal episodes, it accounts for more than half the days with diarrhea [5]. LDD has been shown to have a higher mortality rate among infants compared to acute diarrhea [5, 6] in addition to associations with decreased cognitive function, delayed growth and nutritional deficiencies [7, 8]. Evidence on health inequalities in diarrhea duration is limited, except for severe episodes, which disproportionately affect children < 5 years and lead to longer illness duration [9].

A systematic review of predictive modeling for diarrhea in pediatric populations, encompassing 38 studies, identified that the most common research topics were disease forecasts (14 studies, 36.8%), vaccine-related predictions (9 studies, 23.7%), and disease/pathogen detection (5 studies, 13.2%) with machine learning (ML) as the primary modelling technique (32%) [10]. The review also highlighted a significant research gap in studying the outcomes of diarrheal illness, including LDD. This gap is exacerbated by inadequate diagnostic capacity in many LMICs, including Kenya, which often prevents clinicians from promptly diagnosing and treating enteropathogens associated with LDD [11]. Developing a highly sensitive model that utilizes socio-demographic and clinical characteristics of patients could facilitate the timely identification of children at heightened risk of LDD by clinicians, enabling better, timelier care and close monitoring potentially improving outcomes among this vulnerable group of children. Beyond developing a model with good predictive accuracy, there is need to assess its transportability in light of dynamic nature of data in healthcare including underlying patterns, trends, and distributions [12]. Consequently, we leverage data from two consecutive enteric studies in Kenya to derive and temporally validate a predictive model designed to identify children at increased risk of LDD using ML algorithms.

## Methods

### Study design

This retrospective study leveraged two de-identified diarrheal datasets: the Vaccine Impact on Diarrhea in Africa (VIDA) study for model development and evaluation; the Enteric for Global Health (EFGH) *Shigella* surveillance study for temporal validation. This analysis focuses on data collected from the Kenya site in both studies. There was no patient and public involvement during the design and implementation of the study.

The study design for VIDA have been described elsewhere [13]; in summary, VIDA was designed to assess diarrheal etiologies, rotavirus vaccine effectiveness, and population-based impact of rotavirus vaccine introduction in children aged 0–59 months residing in censused populations in 3 African countries. Moderate-to-severe diarrhea (MSD) cases, defined as children aged 0–59 months presenting at a sentinel health center with diarrhea (defined as ≥ 3 looser-than-normal stools within 24 h) that began within the past 7 days after ≥ 7 diarrhea-free days and had ≥ 1 of the following: sunken eyes, poor skin turgor, dysentery, required intravenous rehydration, or hospitalization. Diarrhea-free controls matched by age, gender and geographical location were enrolled within 14 days of case enrolment. We utilized data from cases enrolled in VIDA over a 36 month period from May 2015 and July 2018.

The EFGH study employed cross-sectional and longitudinal study designs to establish incidence and consequences of *Shigella* medically attended diarrhea (MAD) within 7 country sites in Africa, Asia, and Latin America [14–16]. Eligible MAD cases were children aged 6–35 months presenting at a sentinel health center with diarrhea (defined as ≥ 3 looser-than-normal stools within 24 h) that began within the past 7 days after ≥ 2 diarrhea-free days. Additional eligibility criteria included: residing within the pre-defined EFGH catchment area; plan to remain at their current residence for at least the next 4 months; legal guardian consenting to child’s participation in the study as well willingness to be followed-up for 3 months post-enrolment; child is not being referred to a non-EFGH facility at the time of screening; and site enrollment cap has not been met. Our study utilized EFGH data collected from 01 August, 2022 and 31 July, 2023.

The VIDA study was conducted across 15 sentinel health centers within the Kenya Medical Research Institute's Health and Demographic Surveillance System in Siaya County, while the EFGH study is ongoing in six of these facilities, selected based on patient volume [17]. In both studies, data on demographic, household-level characteristics, illness history, anthropometric and clinical characteristics were collected at enrollment by study research staff. Data from the two enteric studies were used in the current analysis as they followed rigorous procedures [13–16], capturing socio-demographic, clinical, and anthropometric data, along with diarrhea duration over 14 days post-enrollment.

### Pre-Processing

We assessed all demographic, socio-economic and clinical characteristics as potential features. We evaluated the missing data patterns and the missing data points in the variables were imputed using the Multiple Imputation by Chained Equations (MICE) package [18].

### Outcome

The outcome variable, LDD, was defined as a diarrheal episode lasting ≥ 7 days congruent with previous research [5, 19].

Diarrhea duration was determined from two data sources. The pre-enrolment duration was based on caregiver’s report. The days of diarrhea reported in this period were considered to be uninterrupted. The post-enrolment duration involved a 14-day follow-up period after enrollment and was extracted from the data reported by caregiver in a memory aid (Figure S1) and diarrhea diary (Figure S2) for VIDA and EFGH, respectively. Additionally, for the EFGH study, if the caretaker did not return the diarrhea diary, the post-enrolment duration was extracted from the week 4 or month 3 follow-up case report form interview if available.

Based on these two sources, we were able to determine the pre-enrolment diarrhea duration covering 7 days before enrolment and the post-enrolment diarrhea duration covering 14 days after enrolment. This period gives a possible duration of 20-days (day of enrolment was captured in both pre-enrolment and post-enrolment duration). During the post-enrolment period, 2 diarrhea free days were considered an end of an episode consistent with previous studies [19–22].

### Predictors

A total of 68 potential predictors, encompassing demographic factors, household characteristics, illness history, and anthropometric and clinical data, were assessed (Table S1). These variables were documented in the non-medical and medical case report forms of the VIDA study.

### Sample Size

The determination of sample size was conducted utilizing a formula developed by Riley et al. [23].

$${\varvec{n}}={\varvec{P}}/({\varvec{S}}-1){\varvec{l}}{\varvec{n}}(1-\frac{{{\varvec{R}}}^{2}{\varvec{c}}{\varvec{s}}}{{\varvec{S}}})$$

where P = Candidate predictor parameters; S = 1- shrinkage; R2cs- Cox-Snell R squared statistic; P was **10** for LDD prediction; desired shrinkage level was ≤ 10% S = 0.9 and R^2^_cs_ is at least 0.1

For LDD prediction:

*n* = P / ((S-1)ln(1-(R^2^_cs_/S))).

*n* = 10/((0.9–1)ln(1-(0.1/0.9))).

*n* = **849 observations.**

The estimated sample size in the development cohort was at least 849 observations.

### Descriptive analysis

We compared patient characteristics of LDD cases versus non-LDD cases. Proportions were reported for categorical variables and either chi-square or Fisher`s exact test were performed as appropriate. Wilcoxon rank sum tests were used to compare continuous variables as appropriate since the Shapiro–Wilk tests showed they did not follow a normally distribution (Age (*p* < 0.001), diarrhea days (*p* < 0.001), vomit days (*p* < 0.001), respiratory rate (*p* < 0.001) and Vesikari score (*p* < 0.001)).

### Feature selection

We conducted feature selection with the goal of optimizing model accuracy, minimizing computational cost and enhancing interpretability of the models. This was implemented using the Boruta package [24], an all relevant feature selection wrapper around the random forest algorithm that selects relevant features by comparing original attributes' importance with importance achievable at random using their permuted copies. Random forest is an algorithm that builds an ensemble of decision trees trained with a bagging approach to get a more accurate and stable prediction [25].Confirmed and tentative features were subsequently used in model development. Although, rectal straining and breastfeeding were confirmed features, they were not included in the model development since the EFGH study used for temporal validation did not collect them. Table 1 presents the final set of 10 predictors used for model development.

**Table 1:** Final Predictors used in model development

*Variable*	*Description*	*Variable Type*	*Levels*
Agegroup	Age of child	Categorical	**1**- 0–11 months; **2**- 12–23 months; **3**- 24–59 months
Vesikari_score	Severity of diarrhea episode based on Modified Vesikari Score	Categorical	**1**-Mild;** 2**- Moderate; **3**- Severe
Diarr_days	Diarrhea days prior to enrolment as reported by caregiver	Numeric	1–7 days
resp_rate	Respiratory rate of child as measured by clinician at enrolment	Numeric	
Vomit_days	Duration of vomiting as reported by caregiver	Numeric	1–7 days
freq_vomit	Frequency of vomiting as reported by caregiver	Categorical	**1**–0;** 2**–1; **3**–2-4; **4**- ≥ 5
Vomit	Vomiting as reported by caregiver	Binary	**1**- Yes; **0**-No
Rotavirus_vacc	Rotavirus vaccination status based on child immunization card	Categorical	**1**- ≥ 1 dose; **0**- 0 doses
Skin_turgor	Skin turgor of child as assessed by clinician during enrolment	Categorical	**1**- Slow/Very slow **0**-Normal
Stool_count	Number of loose stools in 24 h as reported by caregiver	Categorical	**1**- 3; **2**- 4–5; **3**- ≥ 6

### Model development and evaluation

**Fig. 1:**
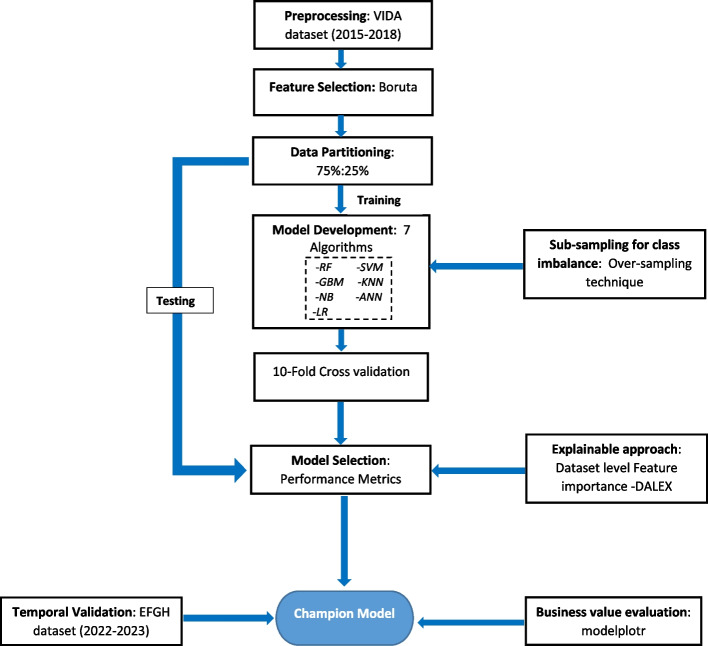
Model development and validation schematic diagram. *RF-Random Forest; GBM-Gradient Boosting; NB- Naïve Bayes; LR-Logistic Regression; SVM- Support vector machine; KNN-K-nearest neighbors; ANN-Artificial Neural Networks, VIDA- Vaccine Impact on Diarrhea in Africa Study, EFGH-Enterics for Global Health Shigella Study

The schematic diagram for model development and validation is shown in Fig. 1. We modeled two scenarios representing two different use cases: i.) the probability of an acute diarrheal episode progressing to LDD based on the child’s signs and symptoms at presentation to hospital ii.) The probability of a diarrheal episode lasting an additional 7 or more days after presentation to hospital (i.e. excluding pre-hospital days of diarrhea).

The first scenario aims to aid healthcare providers in early identification and better management of children at increased risk of LDD. The second scenario hopes to address possible caretaker concerns on how long their child’s diarrheal episode would last from the day of medical treatment. To build the LDD prediction model, we applied 7 ML algorithms including: Random Forest (RF), Gradient Boosting (GBM), Naive Bayes (NB), Logistic regression (LR), Support vector machine (SVM), K-nearest neighbors (KNN) and Artificial Neural Networks (ANN). The algorithms were implemented in R version 4.2.2 using the Caret package [26].

We performed split-sampling by conducting a 75:25% data split to partition the development data (VIDA) into training and test sets [27]. The partitioned data for model training (*n* = 1,112) exceeded the calculated sample size. The LDD predictive models were then developed in the training dataset. To ensure robust evaluation and tuning of these models, we employed tenfold cross-validation [28, 29] to obviate under-fitting or overfitting of the model. We employed over-sampling technique [30] within the resampling procedure to handle the modest class imbalance in our target variable (LDD) since a disparity in the frequencies of the observed classes can have a significant negative impact on model fitting. The summary of the hyper-parameters assessed for each algorithm, along with the best hyper-parameters identified during the grid search are shown in Table S2. The models generated probability estimates, which were classified as LDD using the default threshold of 0.5. The models from the training data were evaluated in the test dataset using the following performance metrics: sensitivity, specificity, positive predictive value (PPV), negative predictive value (NPV) and F1-score. Receiver operating characteristic (ROC) curves were constructed and the area under the curve (AUC) and the precision-recall area under the curve (PRAUC) for each model was computed using the precrec package [31].

We assessed calibration in the built models using Brier scores (the mean squared error between the actual outcome and the estimated probabilities), Spiegelhalter’s *z*-test (a formal measurement that serves as a proxy for calibration calculated from the decomposition of Brier score) and its accompanying *p*-value [32]. The champion model was the best predictive model from the pool of developed models based on the AUC metric. We plotted the calibration plot for the champion model.

We conducted explanatory model analysis (EMA) for the top four models using a model agnostic procedure to estimate SHapley Additive exPlanations (SHAPs) attributions. This was implemented using the DALEX package [33]. The SHAP values were plotted as bar plots in descending degree of importance with the red color signifying a negative association and green color showing a positive association. We further conducted temporal validation on the champion model to assess its transportability and generalizability [34]. Due to the difference in case definition between the two studies, we also conducted a sensitivity analysis of the temporal validation using a subset of EFGH participants who met the VIDA inclusion criteria.

To evaluate the business value of the predictive model, modelplotr package [35] was used to build valuable evaluation plots (cumulative gains, cumulative lift, response and cumulative response plots). The cumulative gains plot was used to visualize the percentage of the target class members that were selected if we decided to select up until percentile X while the cumulative lift plot was used to explain how much better selecting based on our model was compared to taking random selections. The response plot was used to plot the percentage of target class observations per percentile. Lastly, the cumulative response plot was used to show the expected percentage of the target class observations in the selection, when we apply the model and select up until percentile X. Descriptive analysis, predictive modelling for LDD and plotting were all performed in R version 4.2.2 [36].

## Results

### Patient characteristics

**Fig. 2:**
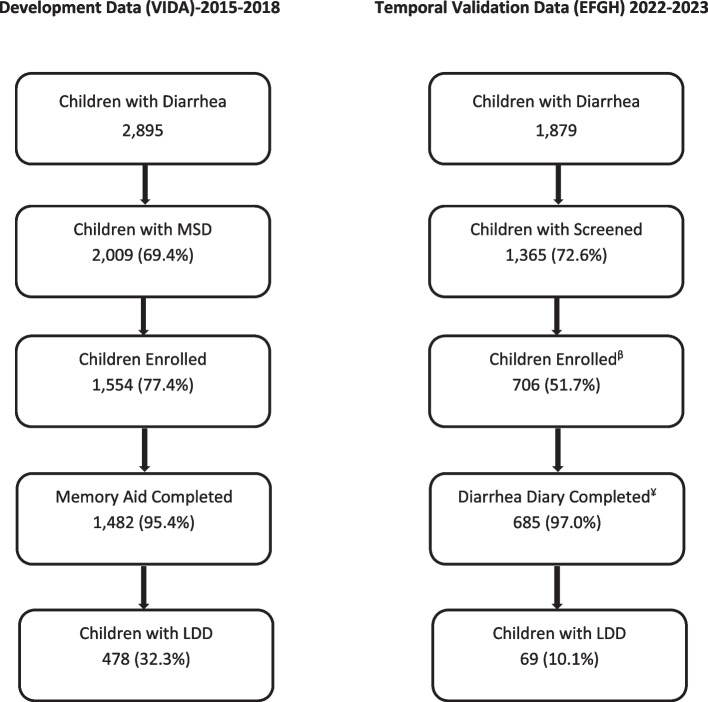
Enrolment flow diagram of diarrhea cases in VIDA (2015–2018) and EFGH (2022–2023). MSD- Moderate-to-severe diarrhea. MAD- Medically attended diarrhea, LDD-Longer duration diarrhea, VIDA-Vaccine Impact on Diarrhea in Africa study, EFGH-Enterics for Global Health Shigella Study.^β−^Children enrolled and successfully followed up at week-4 or have surpassed the upper limit for week-4 follow-up (≥ 67 days post enrolment). ^¥−^Diarrhea duration obtained from Follow-up form where diarrhea diary was not returned but follow-up data was available

During VIDA (development dataset), 2,895 children aged < 5 years sought care for diarrhea in the sentinel health centers, of whom 2,009 (69.4%) had MSD and 1,554 (77.4%) met the study case definition and were subsequently enrolled. Among those enrolled 1, 482 (95.4%) had their memory aids completed by the caretakers, of whom 478 (32.3%) had LDD. While in EFGH (temporal validation dataset), 1,879 children aged < 5 years sought care for diarrhea in the SHCs, of whom 1, 365 (72.6%) were eligible for screening and 706 (51.7%) met the study case definition and were subsequently enrolled. Among those enrolled 685 (97.0%) had their diarrhea diaries completed by the caretakers, of whom 69 (10.1%) had LDD (Fig. 2). There was a statistically significant difference in prevalence of LDD between VIDA and EFGH studies (478 [32.3%] vs 69 [10.1%]; *p* < 0.001). Additionally, we observed significant differences in the baseline characteristics of participants in the two studies. Specifically, compared to EFGH participants, VIDA participants were older (Median age in months [IQR]: 15.0 [9.0–25.0] vs 13.6 [8.9–20.4], *p* = 0.0361), had more severe diarrheal episodes (Median Vesikari score [IQR]: 10 [8–13] vs 8 [6–10], *p* < 0.001), had a higher respiratory rate (Median [IQR]: 36.5 [31.5–41.0] vs 33.0 [28.0–39.0], *p* < 0.001).

Moreover, VIDA participants were more likely to present with vomiting (843 [56.9%] vs 346 [50.5%], *p* = 0.006), decreased skin turgor (614 [41.4%] vs 137 [20.0%], *p* < 0.001) and severe dehydration (388 [26.2%] vs 22 [3.2%], *p* < 0.001) compared to EFGH participants (Table S3).

The characteristics of VIDA participants stratified by LDD status are shown in Table 2. Children who had LDD were younger than those who did not (Median age in months [IQR]: 13 [7-20] vs 16 [10-27], *p* < 0.001). Furthermore, compared with those who did not have LDD, those with LDD had a higher respiratory rate (Median [IQR]: 37.5 [33–42.5] vs 36 [31-40], *p* < 0.001), and a higher Vesikari score (Median [IQR]: 11 [9-13] vs 10 [8-12], *p* < 0.001). Additionally, caretaker education, breastfeeding, stool frequency in 24 h, belly pain, rectal straining, cough, number of vomiting episodes, prior home oral rehydrating salts use, rotavirus vaccination, fast breathing and decreased skin turgor were significantly associated with LDD.

The distribution of LDD cases across the development, internal validation and temporal validation datasets was 359/1,112 (32.3%), 119/390 (32.2%) and 69/685 (10.1%), respectively.

**Table 2:** Characteristics of children aged < 5 years seeking care for moderate-to-severe diarrhea in Kenya stratified by Diarrhea duration, 2015–2018

	Longer Duration Diarrhea (LDD)		
**Characteristics (*N* = 1,482)**	**Yes (*n* = 478)**	**No (*n* = 1,004)**	***p*-value**
	**n (%)**	**n (%)**	
**Demograhic**			
Median age [IQR]	13 [7-20]	16 [10-27]	** < 0.0001**
Age Category			
0–11 months	225 (40.4)	332 (59.6)	** < 0.0001**
12–23 months	152 (29.9)	356 (70.1)	
24–59 months	101 (24.2)	316 (75.8)	
Gender: Female	213 (31.5)	463 (68.5)	0.574
**Household Details**			
Caretaker education (> = Secondary) (*n* = 1,481)	53 (25.7)	153 (74.3)	**0.03**
Natural Floor (*n* = 1,481)	323 (33.4)	644 (66.6)	0.177
Refined/Electric Primary Fuel Source (*n* = 1,478)	14 (24.1)	44 (75.9)	0.183
**Clinical characteristics**			
***By History***			
Breastfeeding before diarrhea onset			** < 0.0001**
None	147 (26.3)	411 (73.7)	
Exclusive	42 (43.3)	55 (56.7)	
Partial	289 (35.0)	538 (65.0)	
Median diarrhea days [IQR]	4 [3-5]	2 [2,3]	< 0.0001
Stool Count			
3	75 (28.1)	192 (71.9)	**0.037**
4–5	256 (31.3)	562 (68.7)	
≥ 6	147 (37.0)	250 (63.0)	
Belly Pain (*n* = 1,427)	299 (34.5)	568 (65.5)	**0.024**
Rectal straining	155 (39.7)	235 (60.3)	** < 0.0001**
Cough	280 (34.7)	526 (65.3)	**0.025**
Vomiting	255 (30.3)	588 (69.7)	0.058
No. of vomit			
0	223 (34.9)	416 (65.1)	**0.026**
1	48 (28.7)	119 (71.3)	
2–4	177 (32.7)	364 (67.3)	
≥ 5	30 (22.2)	105 (77.8)	
Median vomit days [IQR]	2 [1-3]	2 [1,2]	** < 0.0001**
Home ORS use	58 (43.0)	77 (57.0)	**0.005**
Rotavirus vaccination (*n *= 1,389)	414 (35.2)	761 (64.8)	** < 0.0001**
***At enrolment***			
Very Thirsty (*n* = 1,465)	350 (33.5)	695 (66.5)	0.1
Fast breathing	63 (39.1)	98 (60.9)	**0.048**
Median Respiratory rate [IQR]	37.5 [33–42.5]	36 [31-40]	** < 0.0001**
Dry mouth			
Normal	4 (14.8)	23 (85.2)	0.053
somewhat Dry	441 (33.1)	891 (66.9)	
Very dry	33 (26.8)	90 (73.2)	
Skin turgor (slow/very slow)	222 (36.2)	392 (63.8)	**0.007**
Mental Status			0.093
Normal	199 (31.3)	437 (68.7)	
Restless/Irritable	266 (32.4)	555 (67.6)	
Lethargic/Unconscious	13 (52.0)	12 (48.0)	
Under Nutrition	65 (37.4)	109 (62.6)	0.125
Vesikari Score			** < 0.0001**
Mild	15 (10.3)	130 (89.7)	
Moderate	204 (32.4)	426 (67.6)	
Severe	259 (36.6)	448 (63.4)	
Median vesikari score [IQR]	11 [9-13]	10 [8-12]	** < 0.0001**
Cipro_ceft	20 (24.4)	62 (75.6)	0.117

^β−^ Includes electricity, propane, butane, natural gas

*ORS*-Oral rehydration solution.

The following variables had a *p*-value ≥ 0.2 and are not included in the table: No. of children < 5 years in households; Total assets; Animal ownership; improved water; improved sanitation; shared facility; stool type; Blood in stool; drinks poorly; unable to drink; fever; restless; lethargy; unconscious; rectal prolapse; difficulty breathing; convulsion; sunken eyes; home zinc use; capillary refill; chest indrawing; sunken eyes; Bipedal edema; Abnormal hair; Dehydration; ORS at facility; Zinc at facility; IV rehydration; any_antibiotic; Malaria diagnosis; Dysentry diagnosis; Stunting; Wasting.

### Feature selection

**Fig. 3:**
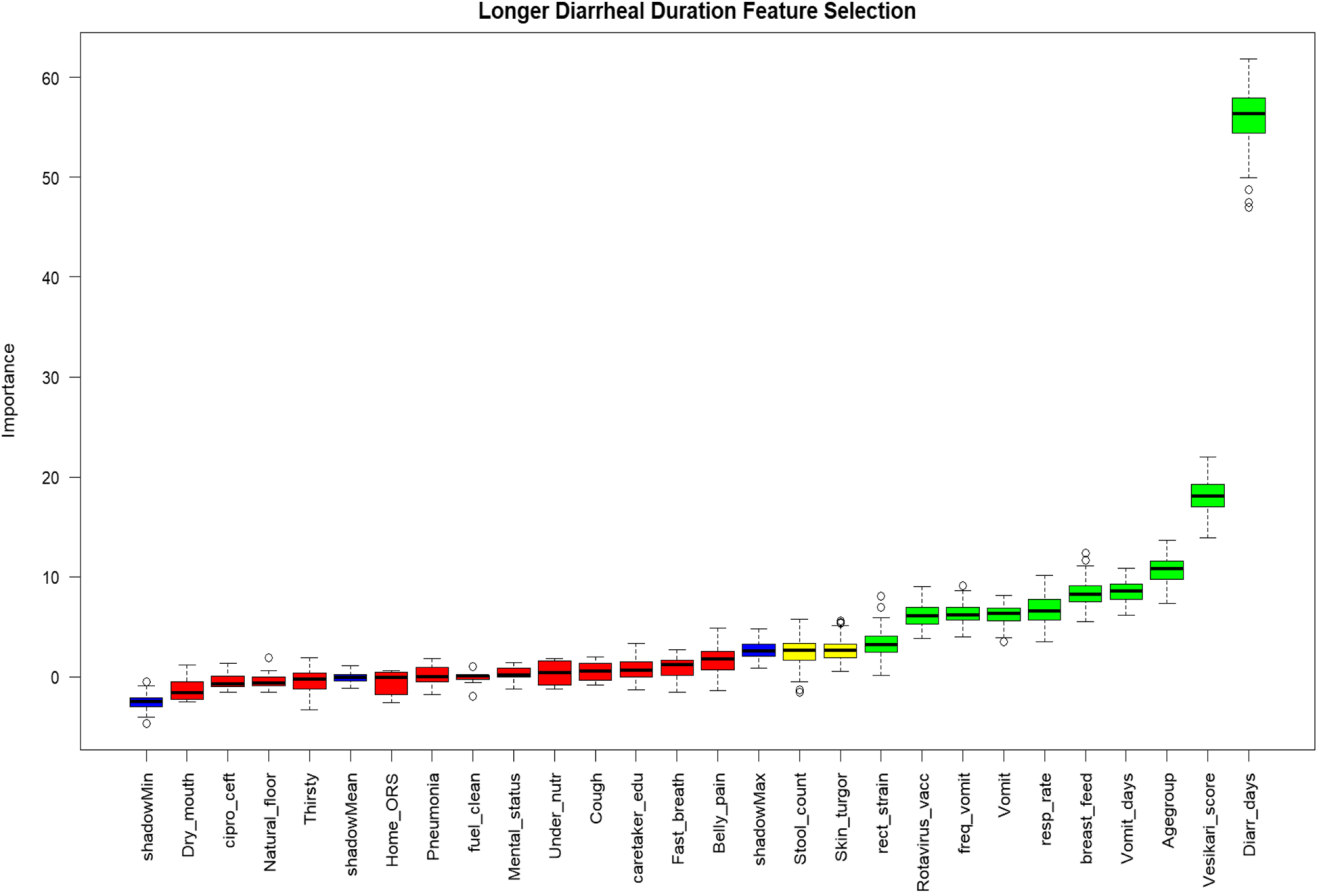
Feature selection for longer duration diarrhea among children aged < 5 years presenting with moderate to severe diarrhea in rural western Kenya, 2015–2023. Green, yellow, red and blue boxplots represent the Z scores of selected, tentative, rejected and shadow features, respectively. Selected and tentative features: *Diarr_days; Vesikari; Agegroup; Vomit_days;breast_feed; resp_rate; Vomit; freq_vomit; Rotavirus vaccination; Rectal straining*; *Stool_count*; *Skin_turgor.*The following additional features were rejected and are not included in the Figure: *No. of children* < *5 years in households; Total assets; Animal ownership; improved water; improved sanitation; shared facility; stool type; Blood in stool; drinks poorly; unable to drink; fever; restless; lethargy; unconscious; rectal prolapse; difficulty breathing; convulsion; sunken eyes; home zinc use; capillary refill; chest indrawing; sunken eyes; Bipedal edema; Abnormal hair; Dehydration; ORS at facility; Zinc at facility; IV rehydration; any_antibiotic; Malaria diagnosis; Dysentry diagnosis; Stunting; Wasting*

From the feature selection analysis, the selected variables in order of importance were diarrhea days prior to presentation (55.1%), Vesikari score (18.2%), age group (10.7%), vomit days (8.8%), breastfeeding (8.4%), respiratory rate (6.5%), vomiting (6.4%), number of vomits in last 24 h (6.2%), rotavirus vaccination (6.1%) and rectal straining (3.4%). Skin pinch (2.4%) and number of loose stools in last 24 h (2.4%) were tentative features (Fig. 3).

### Model performance

We evaluated seven ML algorithms in the prediction of LDD. From the developed models, sensitivity was highest in the RF model (80.7%), followed by the LR (76.5%), ANN (75.6%), SVM (73.9%), KNN (73.1%), the GBM model (72.3%) and lowest in the NB model (69.7%). The specificity of the GBM and SVM models were the highest (76.5%), followed by ANN (74.9%), LR (74.5%), RF and NB (74.1%), and lowest in the KNN model (72.1%). The PPV ranged between 55.4%—59.9% while the NPV ranged between 83.8%—89.0%. The AUC of the models in decreasing order was 83.0%, 82.0%, 81.5%, 81.1%, 80.5%, 79.7% and 77.3% for RF, SVM, ANN, GBM, LR, KNN and NB, respectively (Table 3). The RF model emerged as the champion model with 80.7%, 74.1%, 59.6%, 89.0%, 68.6%, 83.0% and 90.0% for sensitivity, specificity, PPV, NPV, F1-score, AUC and PRAUC, respectively.

**Table 3:** Longer Duration Diarrhea (LDD) prediction models with Over-sampling technique used in the resampling procedure: Model Performance

	***LDD Prediction with over-sampling technique***						
**Algorithm**	**Sensitivity % [95% CI]**	**Specificity % [95% CI]**	**PPV % [95% CI]**	**NPV % [95% CI]**	**F1-Score % [95% CI]**	**AUC % [95% CI]**	**PRAUC % [95% CI]**
RF	80.7 [72.4–87.3]	74.1 [68.2–79.4]	59.6 [51.6–67.3]	89.0 [83.9–92.9]	68.6 [56.3–75.3]	83.0 [78.6–87.5]	90.0 [85.9–93.8]
GBM	72.3 [63.3–80.1]	76.5 [70.8–81.1]	59.3 [50.8–67.4]	85.3 [80.0–89.7]	65.2 [41.7–73.1]	81.1 [76.3–86.0]	87.8 [83.3–93.0]
NB	69.7 [60.7–77.8]	74.1 [68.2–79.4]	56.1 [47.7–64.2]	83.8 [78.3–88.4]	62.2 [36.4–68.9]	77.3 [72.1–82.6]	85.7 [82.2–89.0]
LR	76.5 [67.8–83.8]	74.5 [68.6–79.8]	58.7 [50.5–66.5]	87.0 [81.7–91.2]	66.4 [44.1–69.4]	80.5 [75.6–85.4]	88.7 [84.4–92.7]
SVM	73.9 [65.1–81.6]	76.5 [70.8–81.6]	59.9 [51.5–67.9]	86.1 [80.9–90.4]	66.2 [42.2–69.6]	82.0 [77.3–86.7]	89.3 [85.1–93.1]
KNN	73.1 [64.2–80.8]	72.1 [66.1–77.6]	55.4 [47.3–63.3]	85.0 [79.5–89.5]	63.0 [40.4–70.7]	79.7 [74.9–84.5]	87.0 [83.9–91.0]
ANN	75.6 [66.9–83.0]	74.9 [69.1–80.1]	58.8 [50.6–66.7]	86.6 [81.4–90.9]	66.1 [46.3–74.1]	81.5 [76.8–86.2]	89.1 [84.8–93.6]

*RF Random Forest, *GBM *Gradient Boosting, *NB* Naïve Bayes, *LR *Logistic Regression, *SVM* Support vector machine, *KNN-K *nearest neighbors, *ANN *Artificial Neural Networks;

*95% CI* 95% Confidence Interval, *PPV* Positive Predictive Value, *NPV* Negative Predictive Value, *AUC* Area under the Curve, *PRAUC* Precision Recall Area under the Curve.

The receiver operating characteristic (ROC) curves for LDD prediction models are shown in Figure S3. Furthermore, in the prediction of the duration of diarrhea post-enrolment (≥ 7 days), the model performance ranged between 42.3%-78.8%, 45.3%-72.3%, 16.8%-22.1%, 88.3%-90.9%, 26.5%-30.7%, 52.9%-64.4%, and 86.9%-92.0% for sensitivity, specificity, PPV, NPV, F1-score, AUC and PRAUC, respectively (Table 4). The model performance in the prediction of LDD when no sub-sampling technique was employed are shown in Table S4.

**Table 4:** Post-enrolment duration (≥ 7 days) prediction models with Over-sampling technique used in the resampling procedure: Model Performance

*Post-enrolment Duration Prediction (*≥ *7 days)*							
**Algorithm**	**Sensitivity % [95% CI]**	**Specificity % [95% CI]**	**PPV % [95% CI]**	**NPV % [95% CI]**	**F1-Score % [95% CI]**	**AUC % [95% CI]**	**PRAUC % [95% CI]**
RF	48.1 [34.0–62.4]	72.3 [67.1–77.2]	22.1 [14.9–30.9]	89.5 [85.1–93.0]	30.3 [-19.4–56.0]	63.3 [55.9–70.7]	91.6 [88.1–94.4]
GBM	42.3 [28.7–56.8]	71.1 [65.7–76.0]	19.3 [12.5–27.7]	88.3 [83.7–92.0]	26.5 [-25.7–55.3]	61.1 [54.1–68.1]	91.7 [87.7–94.8]
NB	78.8 [65.3–88.9]	45.3 [39.7–50.9]	19.1 [14.0–25.0]	92.9 [87.7–96.4]	30.7 [0.5–45.2]	62.3 [54.6–70.0]	90.6 [87.6–92.9]
LR	61.5 [47.0–74.7]	59.1 [53.5–64.6]	19.8 [13.9–26.7]	90.4 [85.5–94.0]	29.9 [-11.7–39.7]	64.2 [57.1–71.3]	91.8 [88.5–95.2]
SVM	59.6 [45.1–73.0]	62.6 [57.0–67.9]	20.7 [14.5–28.0]	90.5 [85.8–94.0]	30.7 [-12.6–50.1]	62.7 [55.7–69.7]	91.2 [87.3–95.3]
KNN	59.6 [45.1–73.0]	51.6 [45.9–57.2]	16.8 [11.7–22.9]	88.6 [83.2–92.8]	26.2 [-12.9–43.5]	52.9 [44.5–61.3]	86.9 [83.7–89.9]
ANN	67.3 [52.9–79.7]	53.5 [47.8–59.0]	19.1 [13.7–25.6]	90.9 [85.8–94.6]	29.8 [-7.8–50.2]	64.4 [57.2–71.6]	92.0 [88.8–95.3]

*RF Random Forest, *GBM *Gradient Boosting, *NB* Naïve Bayes, *LR *Logistic Regression, *SVM *Support vector machine, *KNN-K *nearest neighbors, *ANN *Artificial Neural Networks.

*95% CI* 95% Confidence Interval, *PPV* Positive Predictive Value, *NPV* Negative Predictive Value, *AUC* Area under the Curve, *PRAUC* Precision Recall Area under the Curve.

### Calibration and Explanatory model analysis

**Fig. 4:**
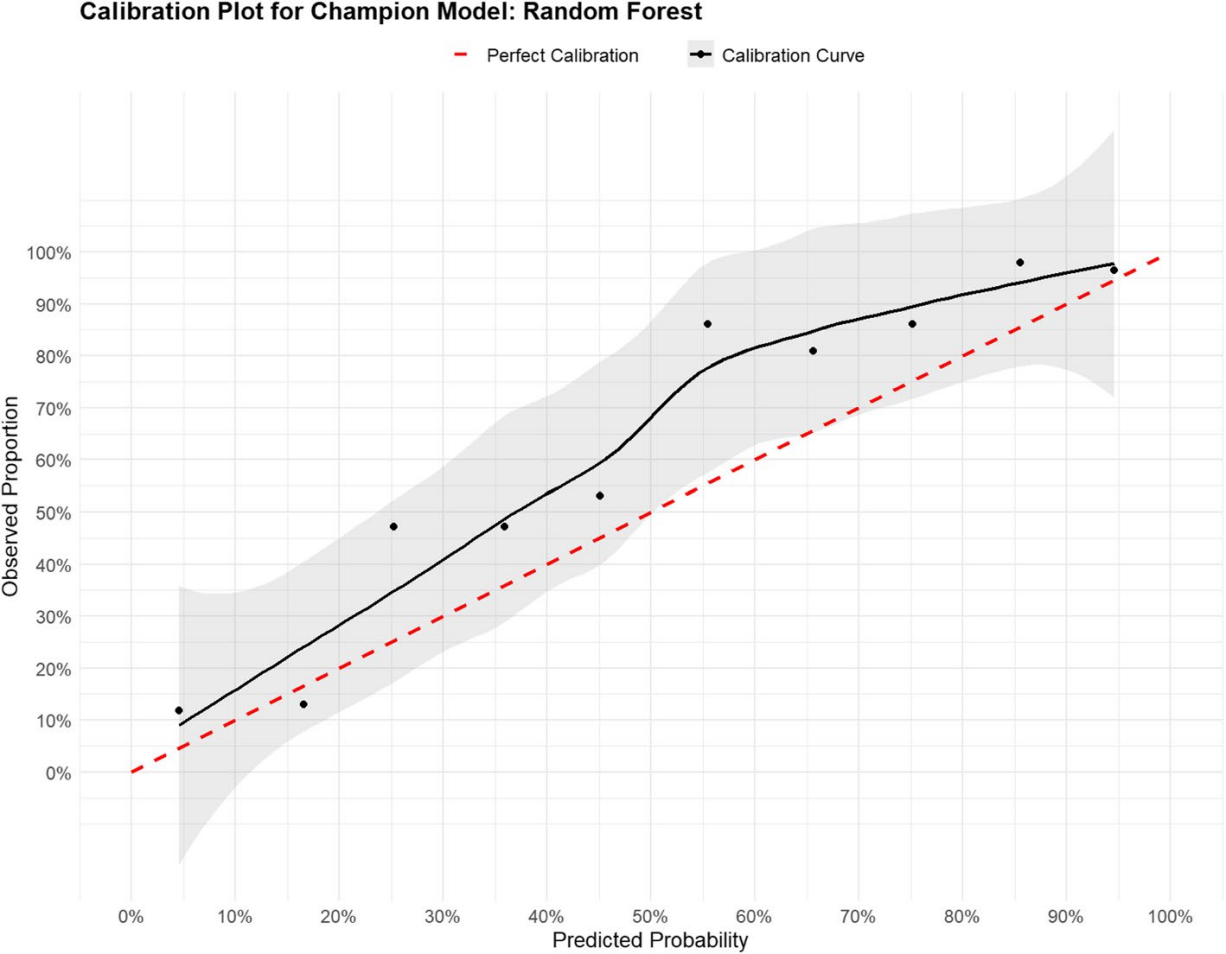
Calibration plot of the random forest champion model

Overall the Brier scores were low and ranged between 0.17–0.21, however the Spiegelhalter’s p-value showed that the NB and KNN models did not calibrate well in the automated algorithm (*p* < 0.05) (Table 5). While the random forest model showed slight deviations from perfect calibration, particularly at the lower end of the predicted probability spectrum (Fig. 4), these deviations were not statistically significant (Spiegelhalter *p*-value = 0.219).

**Table 5:** Calibration results of Longer Duration Diarrhea (LDD) prediction models

*Algorithm*	*Brier Score*	*Spiegelhalter Z-score*	*Spiegelhalter p-value*
RF	0.17	-1.23	0.219
GBM	0.18	-0.95	0.341
NB	0.20	4.82	** < 0.0001**
LR	0.18	-0.58	0.565
SVM	0.17	-0.92	0.357
KNN	0.19	-3.86	** < 0.0001**
ANN	0.18	-0.18	0.855

**Fig. 5:**
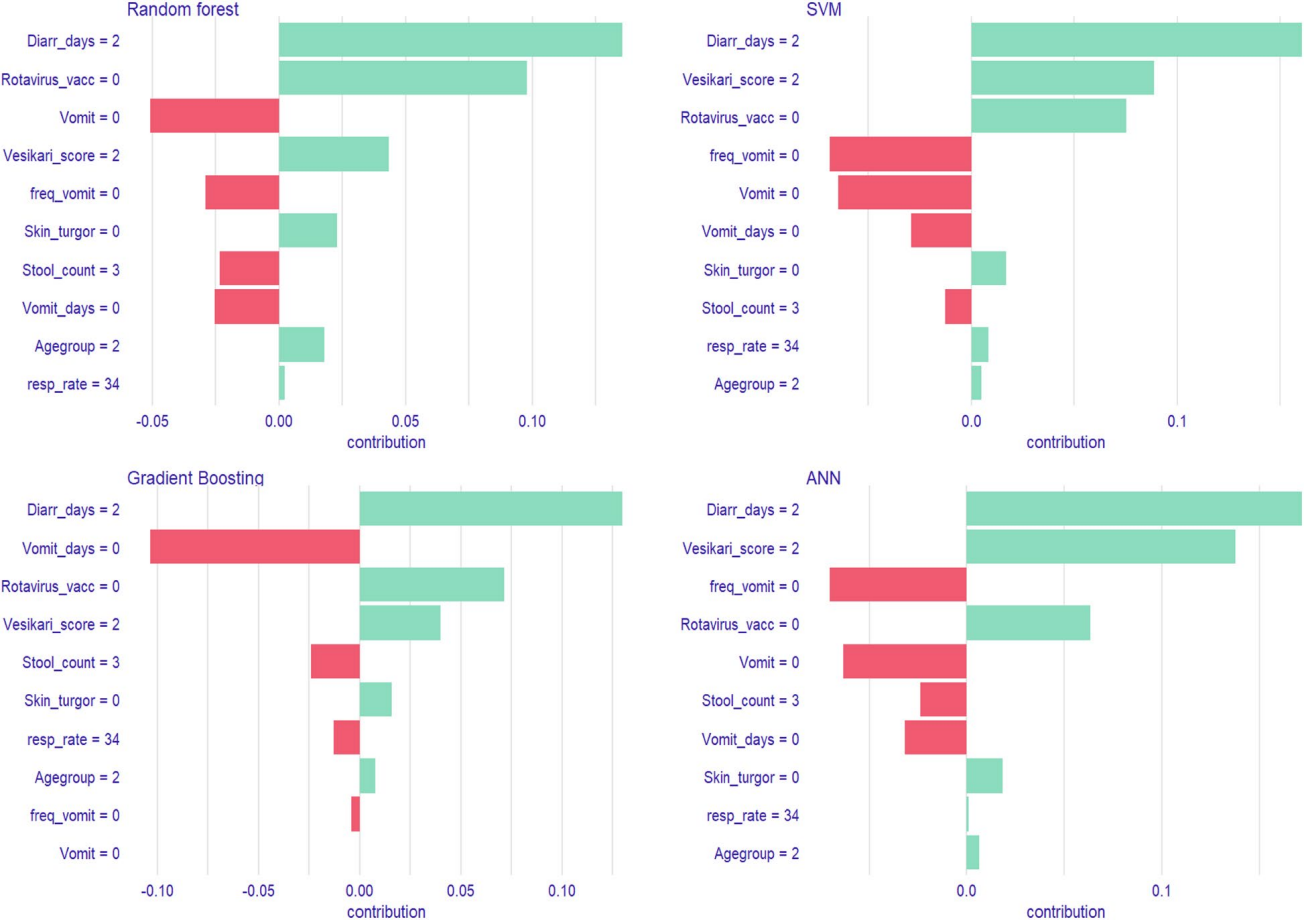
SHAP attributions for the Top 4 Longer Duration Diarrhea (LDD) models. * SVM- Support vector machine; ANN-Artificial Neural Networks. “ Diarr_days = 2”- Pre-enrolment diarrhea days = 2; “Rotavirus_vacc = 0”- No dose of rotavirus vaccine administered; “Vomit = 0”- No vomiting; “Vesikari_score = 2”- Moderate severity of diarrheal disease; “freq_vomit = 0”-maximum number of vomiting = 0; “Skin turgor = 0”- Normal skin turgor; “Stool_count”- ≥ 6 loose stools per day; “Vomit_days = 0”-0 vomiting days; “Agegroup = 2”- 12–23 months

*RF Random Forest, *GBM *Gradient Boosting, *NB* Naïve Bayes, *LR *Logistic Regression, *SVM* Support vector machine, *KNN-K *nearest neighbors, *ANN *Artificial Neural Networks;

From the explanatory model analysis of the prediction of LDD, the degree of importance varied across models with the likelihood of developing LDD increasing with pre-enrolment diarrhea days, severity based on the modified Vesikari score, no rotavirus vaccination, normal skin turgor and age. Conversely, the likelihood of progressing to LDD decreased with no vomiting (vomit = 0, number of vomits in last 24 h = 0, and vomit days = 0) and number of loose stools in last 24 h (≥ 6) (Fig. 5).

**Fig. 6:**
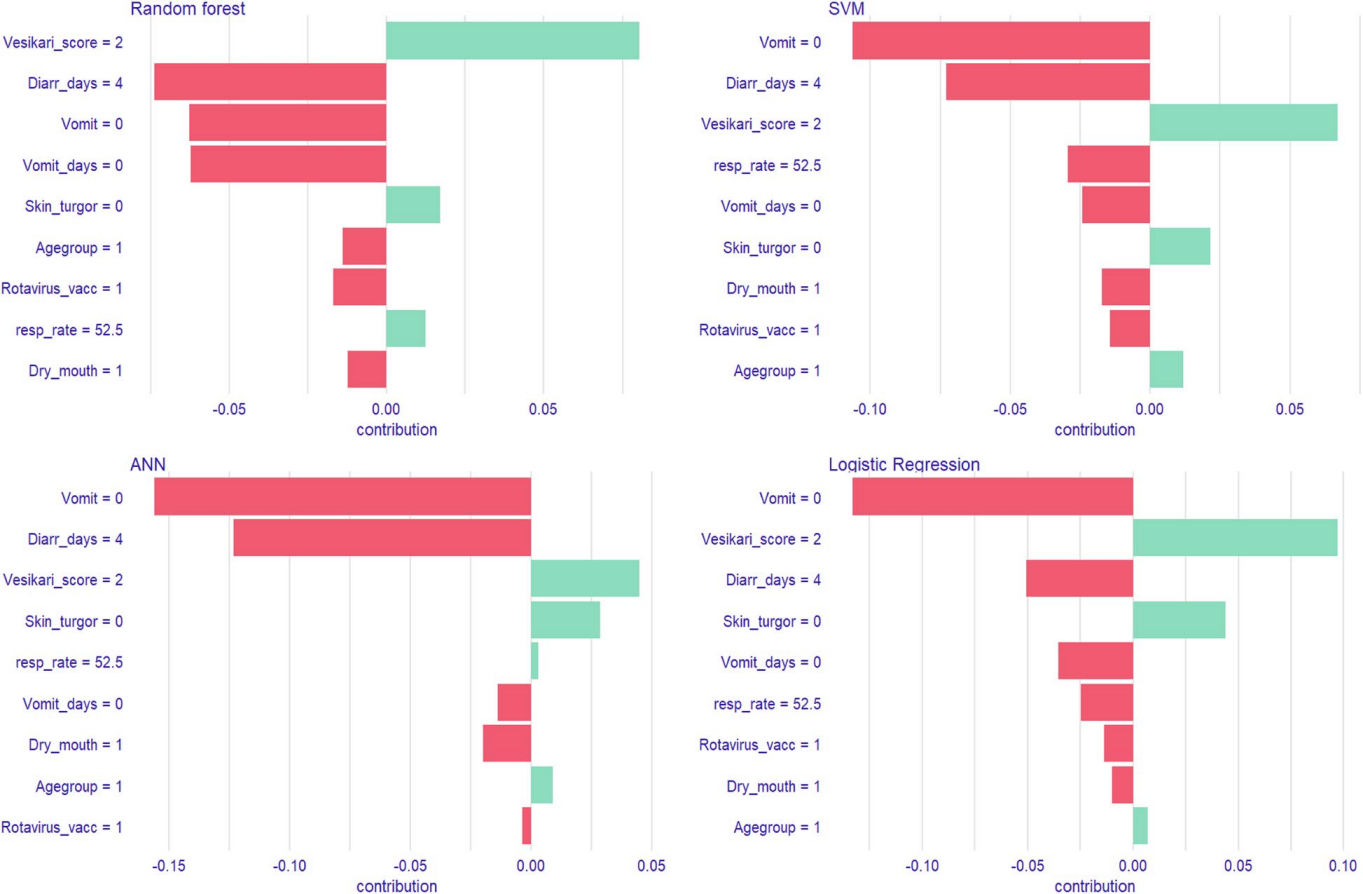
SHAP attributions for the Top 4 Post-enrolment duration (≥ 7 days) models. “Vesikari_score = 2”- Moderate severity of diarrheal disease; “Diarr_days = 4”- Pre-enrolment diarrhea days = 4; “Vomit = 0”- No vomiting; “Vomit_days = 0”-0 vomiting days; “Skin turgor = 0”- Normal skin turgor; “Agegroup = 1”- 0–11 months; “Rotavirus_vacc = 1”- at least 1 dose of rotavirus vaccine administered; “Dry mouth = 1”- Somewhat dry mouth;

Furthermore, we observed similar patterns in the EMA results of the prediction of ≥ 7 days of diarrhea post-enrolment with the only difference being in pre-enrolment diarrhea days, which decreased the likelihood of developing the outcome in this prediction (Fig. 6).

### Business value evaluation

**Fig. 7:**
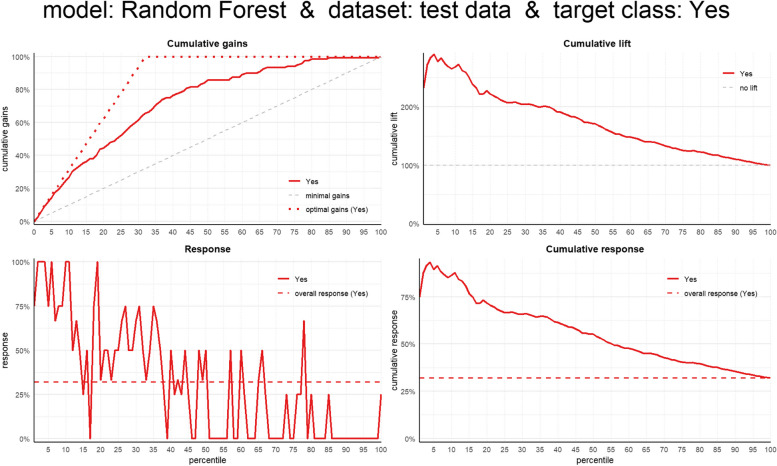
Business value plots for the Random Forest (RF) Model for Longer diarrhea duration (LDD)

From the business value evaluation of our champion model (RF), the cumulative gains plot shows that the model is able to select 46% of the target class (LDD) if we select the top-20% cases based on our model. Additionally, from the cumulative lift plot, our champion model is able to identify 2.6 times more LDD cases compared to a random selection if we pick the top-20% observations based on model probability. Lastly, from the cumulative response plot, 72% of observations in the top-20% cases based on model probability belong to the target class (Fig. 7).

### Temporal validation in EFGH data

**Fig. 8:**
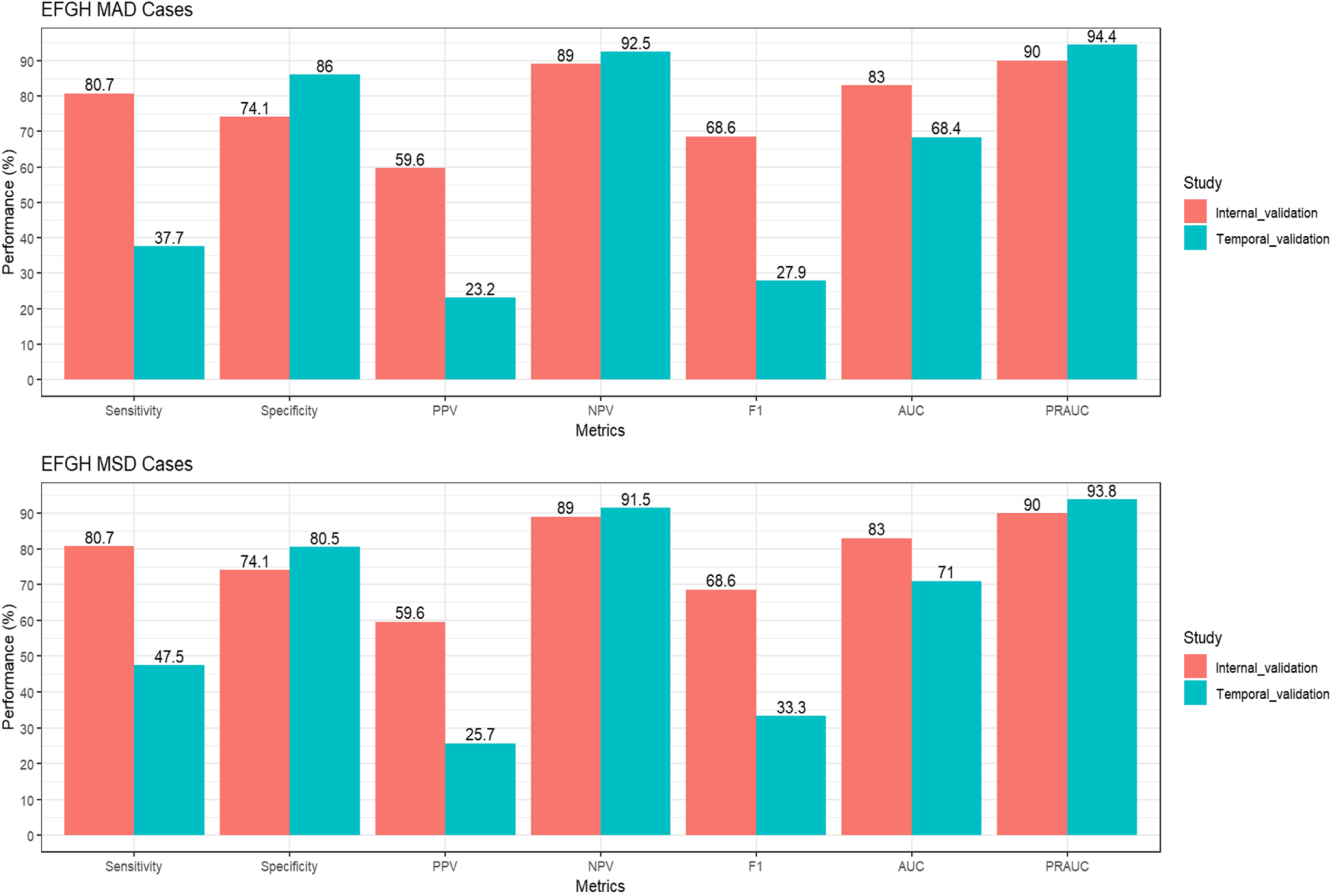
Performance of champion model in development (2015–2018) and temporal validation (2022–2023) datasets. PPV- Positive Predictive Value; NPV- Negative Predictive Value; AUC- Area under the Curve; PRAUC- Precision Recall Area under the Curve

We observed a decline in model performance on the temporal validation dataset, the RF model achieved 37.7%, 86.0%, 23.2%, 92.5%, 27.9%, 68.4% and 94.4% for sensitivity, specificity, PPV, NPV, F1-score, AUC and PRAUC, respectively. We observed a marginal increase in model performance in the sensitivity analysis when including only EFGH enrollees that met the VIDA inclusion criteria, the RF model achieved 47.5%, 80.5%, 25.7%, 91.5%, 33.3%, 71.0% and 93.8% for sensitivity, specificity, PPV, NPV, F1-score, AUC and PRAUC, respectively (Fig. 8).

## Discussion

As the field of machine learning advances, its potential to revolutionize healthcare practices for the benefit of both patients and healthcare systems globally is becoming increasingly evident. This study evaluated the feasibility of ML algorithms in the prediction of LDD among pediatric patients presenting with diarrhea. From our evaluation of 7 ML algorithms, the models achieved good performance with the RF model emerging as the champion model in predicting LDD. However, on the temporal validation data the champion model (RF) did not perform optimally registering a drop of 12.0% in the model AUC, largely driven by a decrease in sensitivity. Moreover, there was a decline in model performance while predicting the probability of having ≥ 7 diarrhea days post-enrolment with the ANN model achieving the best performance. These declines in model performance are likely to be attributable to differences in the study populations [37, 38], with our temporal validation set having fewer LDD episodes and including children with less severe diarrhea. However, we cannot exclude the possibility that some degree of model over-fitting may have contributed to the decrease in performance in our validation dataset. Despite these decreases in performance the models retained good negative predictive values, suggesting they may aid clinicians identify which children are unlikely to experience LDD.

Based on our feature selection, the variables identified as predictors of LDD were diarrhea days, Vesikari score, age group, vomit days, breastfeeding, respiratory rate, vomiting, number of vomits in last 24 h, rotavirus vaccination, rectal straining, skin pinch and number of loose stool in last 24 h. These variables have been documented as risk factors for LDD in previous studies. Specifically, severity of diarrheal disease was an important predictor of LDD in our results with the overall severity (modified Vesikari score) as well as individual elements of the severity score (diarrhea days, vomiting, vomit days, number of vomits in last 24 h, number of loose stool in last 24 h, skin pinch) being predictive of LDD. Our findings are consistent with those of Lima and Guerrant who found in their review that episodes of longer duration were more severe at presentation [39, 40]. Severe diarrheal episodes may cause intestinal inflammation which could lead to prolonged illness and recovery time [41]. Additionally, severe diarrhea through vomiting and high passage of loose stools may lead to significant loss of fluids and electrolytes, which may exacerbate the illness and lead to extended duration of illness as the body needs more time to replenish lost fluids and restore its normal balance of electrolyte. A stronger host immune response to etiologic agents of diarrhea may also lead to more severe symptoms and an extended illness duration.

We also observed younger children were at increased risk of LDD. This finding is similar to findings from previous studies [5, 40, 42] and can possibly be explained by the fact that previous exposure to enteric pathogens which can induce specific immunity that may reduce diarrheal duration and frequency is likely to be minimal in infants and toddlers compared to older children. Additionally, as children age their immune system undergoes development throughout early childhood thereby reducing their vulnerability to infection by microbial agents. We also observed lack of rotavirus vaccination to be a predictor of LDD. Despite the lower vaccine effectiveness of rotavirus vaccine reported in developing countries compared to the developed countries [43], lack of rotavirus vaccination exposes children to severe dehydrating diarrhea that would possibly lead to prolonged duration of illness.

Post-enrolment diarrhea duration (≥ 7 days) was much harder to predict than overall LDD, and we observed a decline in model performance with a difference of up to -26.8% reported in model AUCs. A number of potential reasons have been advanced in literature for poor performance of machine learning algorithms: outliers in the development dataset, class imbalance, overfitting or underfitting, use of less than ideal metric in assessing performance and the data doesn’t represents a predictable pattern [44]. Our model development strategy that involved split sampling, K-fold cross validation and over-sampling technique addresses most of these challenges leaving data that does not represent a predictable pattern as a possible reason for the sub-optimal results observed. The EMA results in the prediction of post-enrolment duration were similar to those of LDD prediction and they showed that severity based on the modified Vesikari score, no rotavirus vaccination, normal skin turgor and age and had a positive effect on the prediction of the outcomes while no vomiting and ≥ 6 loose stools had a negative effect on the prediction of the outcomes. The primary difference between the two outcomes was inclusion of pre-enrolment diarrhea days which would be known to the models and clinicians at presentation.

Approximately, 32 and 10 in every 100 children with MSD and MAD, respectively, develop LDD. This burden coupled with lack of adequate diagnostic capacity [45] and overburdened healthcare workers [46] underscore the need of alternative strategies such as clinical predictive models in prioritizing resources at high-risk children and ensuring close monitoring and better management while allowing low-risk children return home earlier. Our results show the potential of ML algorithms in the rapid identification of at-risk children. This model could be deployed as a web-based application using platforms such as R-shiny or plumber [47, 48], or it could be integrated into electronic medical records systems [49] ensuring it is aligned with clinical workflows. Additionally, implementing a real-time strategy to handle missing predictor values is essential to maximize the model's utility. Furthermore, ensuring logical consistency and identifying outliers through data validation checks on input data can significantly enhance data quality and bolster model implementation. Such simple and flexible deployment methodologies can allow rapid adoption of the model in clinical practice helping to complement clinician judgement in the timely identification of at-risk patients. Moreover, implementing the model will require clinicians to input predictor values, enabling the model to generate a risk profile for the child along with SHAP attributions to enhance interpretability and support informed decision-making. Users will need a basic understanding of the predictors, their clinical significance, and the ability to interpret probability estimates and SHAP attributions. However, further work is needed to address the drop in sensitivity during temporal validation that was probably caused by the baseline differences in severity of disease and possible shift in study population over time. This also suggests that different criteria for predicting LDD could be used across settings with varying severity. Our findings highlight the need for monitoring and periodic retraining of the model in order to maintain its predictive performance. It may also be possible that strengthening laboratory capacity, allowing inclusion of biological data into predictive framework, is an alternative pathway to improve the accuracy of clinical judgements.

In spite of using a robust strategy in model derivation and validation (internal and temporal), our study still has some limitations. While certain pathogens have been shown to be associated with extended duration of diarrhea, we did not use laboratory results although they were available in both studies used. The rationale for this decision was that culture and molecular diagnostic testing is not routinely done in most health facilities and therefore results would be unavailable in the absence of study support hence this data would be missing when using the tool. Additionally, while this model may help to rapidly identify children at increased risk of LDD, no evidenced-based treatment for LDD exists leaving only empiric treatment such as general supportive care and nutritional rehabilitation as possible therapeutic options whenever zinc fails to reduce diarrheal duration. Lastly, as this study utilized data from previous enteric studies not originally designed to evaluate LDD, certain predictors, such as a history of prior LDD, may have been missed. This omission could potentially limit the predictive power of the models. Future research should focus on externally validating these models as well as assessing the potential acceptability and potential impacts of ML models on clinical practice and patient outcomes as well as the cost-effectiveness of such model deployment.

## Conclusions

Our study shows the practical utility of machine learning algorithms in rapid identification of children at increased risk of LDD in our setting. The use of our validated RF model in clinical settings to complement clinician judgement could help to prioritize resources at high-risk children and ensure close monitoring and better management while allowing low-risk children return home earlier. However, successful implementation and widespread adoption will require further research, collaboration, and ethical diligence. There is need to explore its integration into clinical decision-making in order to translate the model outputs into actionable insights and real-world impact.

## Supplementary Information

Supplementary Material 1.

## Data Availability

The data used for the modelling in this study belongs to KEMRI and restrictions apply to the availability of these data. Data cleaning, pre-processing and model development were done in R version 4.1.2. The programming code for R is available on GitHub: https://github.com/bogwel/Prolonged_diarrhea_Prediction.

## Abbreviations

LMICs: Low- and middle-income countries
LDD: Longer duration diarrhea
ML: Machine learning
VIDA: Vaccine Impact on Diarrhea in Africa study
EFGH: Enterics for Global Health
MSD: Moderate-to-severe diarrhea
MAD: Medically attended diarrhea
MICE: Multiple Imputation by Chained Equations
RF: Random Forest
GBM: Gradient Boosting
NB: Naive Bayes
LR: Logistic regression
SVM: Support vector machine
KNN: K-nearest neighbors
ANN: Artificial Neural Networks
PPV: Positive predictive value
NPV: Negative predictive value
ROC: Receiver operating characteristic curves
AUC: Area under the curve
PRAUC: Precision-recall area under the curve
EMA: Explanatory model analysis
SHAPs: SHapley Additive exPlanations
KEMRI SERU: Kenya Medical Research Institute Scientific and Ethical Review Unit
