# Supplementary material for: Derivation and validation of a clinical predictive model for longer duration diarrhea among pediatric patients in Kenya using machine learning algorithms
Source: BMC Med Inform Decis Mak. 2025 Jan 15;25:28. doi: 10.1186/s12911-025-02855-6 (PMC11737202; doi:10.1186/s12911-025-02855-6)
Supplement: Supplementary file 1 — Supplementary Material 1. [file 12911_2025_2855_MOESM1_ESM.pdf]

Supplemental Appendix

Figure S1. VIDA Memory Aid Sheet

Figure S2. EFGH-Shigella Diarrhea Diary

Figure S3. Receiver operating characteristic curve for LDD prediction models

Table S1: List of potential predictors in the development cohort

Table S2: Hyper parameter evaluation

Table S3: Comparison of baseline characteristics among pediatric patients in development and temporal validation datasets.

Table S4. Longer Duration Diarrhea (LDD) prediction models with no sub-sampling technique used in the resampling procedure: Model Performance

VIDA CRF 09A - MEMORY AID SHEET

Site	Center	Child ID	Day	Month	Year
					20

Please complete this form every day for each of the next 14 days. *Kiyie to ndik obokeni pile ka pile e ndalo 14 mabiro.*

1. Each morning when you wake up, decide whether your child had *diarrhea* the previous day. *Diarrhea* means that your child passed 3 or more loose or watery stools that were not normal for him or her. *Pile pile kichiew gokinyi onego one irang ka nyathini ni gi diep odiochieng manyoro.Diep nyiso ni nyathi ne odhi oko mdidek kata moloyo kanyo kata oko ma reprep motimo pi ma ogogore gi kaka odhi ga pile.*
2. Go to the correct day. "☼" means the day you went to the health center or hospital, called "day (1)". The next day is "☼☼", or "day (2)", or and so on. A day begins when you wake up in the morning and ends when you wake up the next morning. *Dhi e odiochieng' makare."☼" manyiso kawuono. "☼☼"nyiso kiny kamano kamano odiochieng' chakore ka ichiew gokinyi kendo rumo ka ichiew okinyi ma luwe.*
3. If your child had diarrhea that day, mark "X" in the dark box under the child with diarrhea for that day [X]. If your child did <u>not</u> have diarrhea, mark "X" in the white box for that day under the child who appears well [X]. **Each day, make only <u>one</u> "X".** *Ka nyathini nigi diep odiochieng'no ket alama X ei box marateng'* [X]. *Ka nyathi onge gi diep ket alama X ei box marachar ei odiochieng'no* [X]. ***ket alama X <u>kamoro achiel</u> e odiochieng' ka odiochieng'.***
4. If you forget for a few days, try to start again on the correct day. *Kapo ni wiwi owil kuom ndalo matin, tem mondo ichak kendo e odiochieng makare.*
5. Keep this form in a safe place. We will come to your house to collect it in about 60 days. *Kan obokeni kama ber wabiro kawe e odi ka ndalo 60 ochopo.*

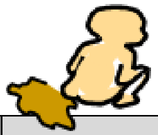

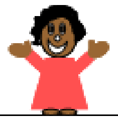

Day *Tarik*			
(1)	☼ (today) *odiochieng' mokuongo*		
(2)	☼☼		
(3)	☼☼☼		
(4)	☼☼☼☼		
(5)	☼☼☼☼☼		
(6)	☼☼☼☼☼☼		
(7)	☼☼☼☼☼☼☼		
(8)	☼☼☼☼☼☼☼☼		
(9)	☼☼☼☼☼☼☼☼☼		
(10)	☼☼☼☼☼☼☼☼☼☼		
(11)	☼☼☼☼☼☼☼☼☼☼☼		
(12)	☼☼☼☼☼☼☼☼☼☼☼☼		
(13)	☼☼☼☼☼☼☼☼☼☼☼☼☼		
(14)	☼☼☼☼☼☼☼☼☼☼☼☼☼☼		

Figure S1. VIDA Memory Aid Sheet

Enterics for Global Health – *Shigella* Surveillance Study (EFGH)
CRF 06— Diarrhea Diary

<table>
<tr><td></td><td></td><td>Day 0 - Previous 24 hours prior to enrollment Visit</td><td>DAY 1 |__|__|-|__|__|- |2|0|2|__| *
*May not be full 24—hour period</td><td>DAY 2 |__|__|-|__|__|- |2|0|2|__|</td><td>DAY 3 |__|__|-|__|__|- |2|0|2|__|</td><td>DAY 4 |__|__|-|__|__|- |2|0|2|__|</td><td>DAY 5 |__|__|-|__|__|- |2|0|2|__|</td><td>DAY 6 |__|__|-|__|__|- |2|0|2|__|</td><td>DAY 7 |__|__|-|__|__|- |2|0|2|__|</td><td>DAY 8 |__|__|-|__|__|- |2|0|2|__|</td><td>DAY 9 |__|__|-|__|__|- |2|0|2|__|</td><td>DAY 10 |__|__|-|__|__|- |2|0|2|__|</td><td>DAY 11 |__|__|-|__|__|- |2|0|2|__|</td><td>DAY 12 |__|__|-|__|__|- |2|0|2|__|</td><td>DAY 13 |__|__|-|__|__|- |2|0|2|__|</td><td>DAY 14 |__|__|-|__|__|- |2|0|2|__|</td></tr>
<tr><td colspan="17">Number of Loose or Watery Stool</td></tr>
<tr><td rowspan="7"></td><td>No loose or watery stool
(Onge Diep)</td><td></td><td></td><td></td><td></td><td></td><td></td><td></td><td></td><td></td><td></td><td></td><td></td><td></td><td></td><td></td></tr>
<tr><td></td><td></td><td></td><td></td><td></td><td></td><td></td><td></td><td></td><td></td><td></td><td></td><td></td><td></td><td></td><td></td></tr>
<tr><td></td><td></td><td></td><td></td><td></td><td></td><td></td><td></td><td></td><td></td><td></td><td></td><td></td><td></td><td></td><td></td></tr>
<tr><td></td><td></td><td></td><td></td><td></td><td></td><td></td><td></td><td></td><td></td><td></td><td></td><td></td><td></td><td></td><td></td></tr>
<tr><td></td><td></td><td></td><td></td><td></td><td></td><td></td><td></td><td></td><td></td><td></td><td></td><td></td><td></td><td></td><td></td></tr>
<tr><td></td><td></td><td></td><td></td><td></td><td></td><td></td><td></td><td></td><td></td><td></td><td></td><td></td><td></td><td></td><td></td></tr>
<tr><td>+</td><td></td><td></td><td></td><td></td><td></td><td></td><td></td><td></td><td></td><td></td><td></td><td></td><td></td><td></td><td></td></tr>
<tr><td colspan="17">Vomiting
(Ng'ok)</td></tr>
<tr><td rowspan="5"></td><td>No vomitting
(Onge ng'ok)</td><td></td><td></td><td></td><td></td><td></td><td></td><td></td><td></td><td></td><td></td><td></td><td></td><td></td><td></td><td></td></tr>
<tr><td></td><td></td><td></td><td></td><td></td><td></td><td></td><td></td><td></td><td></td><td></td><td></td><td></td><td></td><td></td><td></td></tr>
<tr><td></td><td></td><td></td><td></td><td></td><td></td><td></td><td></td><td></td><td></td><td></td><td></td><td></td><td></td><td></td><td></td></tr>
<tr><td></td><td></td><td></td><td></td><td></td><td></td><td></td><td></td><td></td><td></td><td></td><td></td><td></td><td></td><td></td><td></td></tr>
<tr><td></td><td></td><td></td><td></td><td></td><td></td><td></td><td></td><td></td><td></td><td></td><td></td><td></td><td></td><td></td><td></td></tr>
</table>

Figure S2. EFGH-Shigella Diarrhea Diary

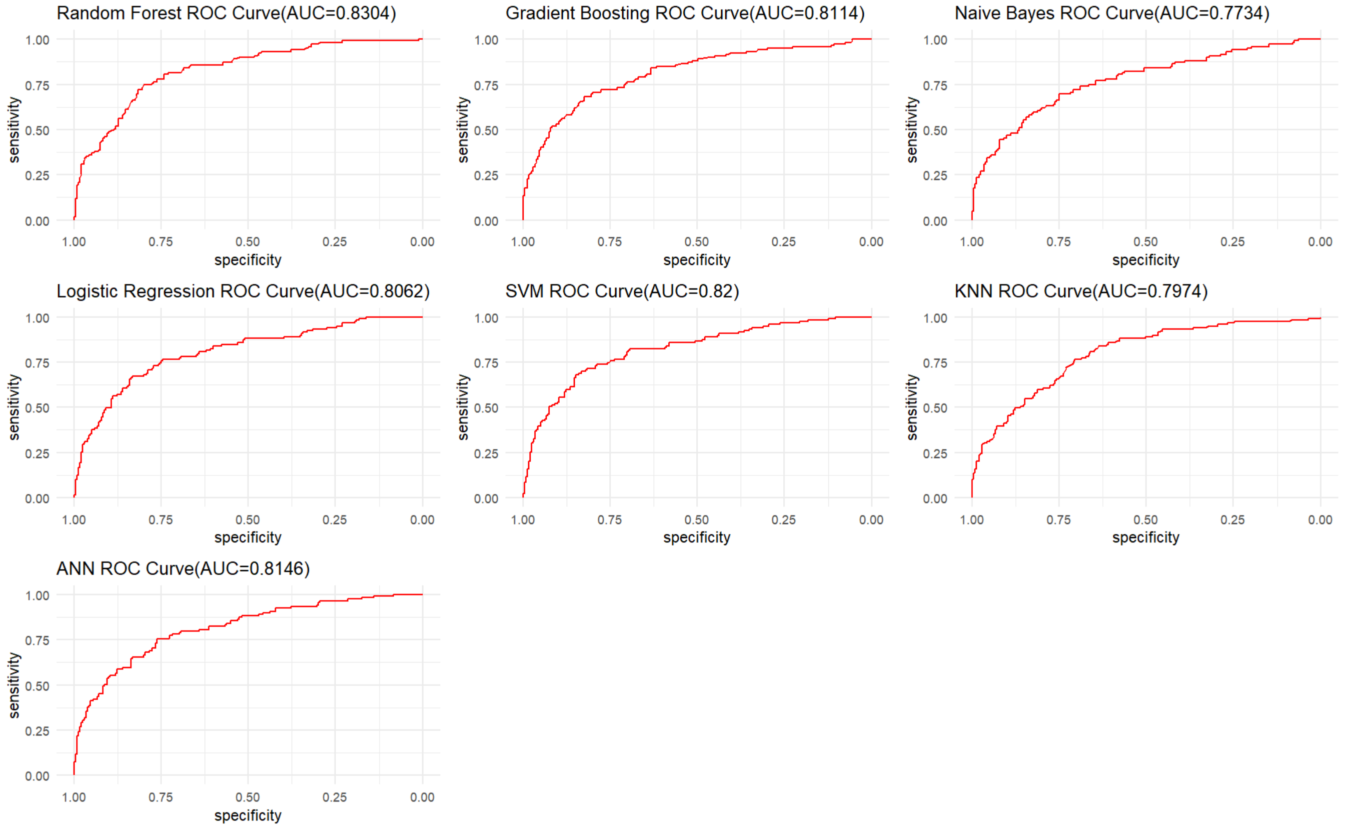

*RF-Random Forest; GBM-Gradient Boosting; NB- Naïve Bayes; LR-Logistic Regression; SVM- Support vector machine; KNN-K-nearest neighbors; ANN-Artificial Neural Networks;
ROC-Receiver operating characteristic curve
AUC-Area under the curve

Figure S3. Receiver operating characteristic curve for LDD prediction models

Table S1: List of potential predictors in the development cohort

Variable	Type	Levels
**Socio-demographic characteristics**		
Age Category	Categorical	**1**- 0-11 months; **2**- 12-23 months; **3**- 24-59 months
Gender:	Binary	1-Male; 2-Female
Caretaker education	Categorical	**1**-<Secondary; **2**-≥Secondary
Number of children < 5 yrs in household	Categorical	**1**- ≤ 2; **2**- ≥ 3
Total Assets	Categorical	**1**- ≤ 3; **2**- ≥ 4
Natural Floor	Binary	**1**- Yes; **0**-No
Refined/Electric Primary Fuel Source	Binary	**1**- Yes; **0**-No
Animal onwership	Binary	**1**- Yes; **0**-No
Improved water	Categorical	**1**- Safely managed; **2**- Basic; **3-** Limited; **4**- unimproved/Surface water
Improved Sanitation	Categorical	**1**- Safely Managed and Basic; **2**- Limited; **3**- unimproved/Open Defecation
Shared facility	Binary	**1**- Yes; **0**-No
**Clinical characteristics**		
**Caregiver reported**		
Breastfeeding before diarrhea onset	Categorical	**0**- None; **1**- Partial; **2**- Exclusive
Pre-enrolment diarrhea days	Numeric	
Stool Type	Categorical	**1**-Simple watery; **2**- Rice watery; **3**- Sticky/Mucoid; **4**- Bloody
Stool Frequency	Categorical	**1**- 3; **2**- 4-5; **3**- ≥ 6
Blood in stool	Binary	**1**- Yes; **0**-No
Very Thirsty	Binary	**1**- Yes; **0**-No
Drinks poorly	Binary	**1**- Yes; **0**-No
Unable to drink	Binary	**1**- Yes; **0**-No
Belly Pain	Binary	**1**- Yes; **0**-No
Fever	Binary	**1**- Yes; **0**-No
Restless	Binary	**1**- Yes; **0**-No
Lethargy	Binary	**1**- Yes; **0**-No
unconscious	Binary	**1**- Yes; **0**-No
Rectal straining	Binary	**1**- Yes; **0**-No
Rectal prolapse	Binary	**1**- Yes; **0**-No
Cough	Binary	**1**- Yes; **0**-No
Difficulty breathing	Binary	**1**- Yes; **0**-No
Convulsion	Binary	**1**- Yes; **0**-No
Vomiting	Binary	**1**- Yes; **0**-No
No. of vomit	Categorical	**1**-0; **2**-1; **3**-2-4; **4**- ≥ 5

Vomit days	Numeric	
**Assessed by Non-medical staff**		
Very Thirsty	Binary	**1**- Yes; **0**-No
Drinks poorly	Binary	**1**- Yes; **0**-No
Sunken Eyes	Binary	**1**- Yes; **0**-No
Wrinkled skin	Binary	**1**- Yes; **0**-No
Restless	Binary	**1**- Yes; **0**-No
Dry mouth	Binary	**1**- Yes; **0**-No
Fast breathing	Binary	**1**- Yes; **0**-No
Home ORS use	Binary	**1**- Yes; **0**-No
Home Zinc use	Binary	**1**- Yes; **0**-No
Rotavirus vaccination	Categorical	**1**- ≥ 1 dose ; **0**- 0 doses
**Clinicain- Assessed**		
Fever (Temp>37.0)	Binary	**1**- Yes; **0**-No
Respiratory rate	Numeric	
Capilary refill (slow/very slow)	Categorical	**1**- Slow/Very slow **0**-Normal
Chest indrawing	Binary	**1**- Yes; **0**-No
Sunken eyes	Binary	**1**- Yes; **0**-No
Dry mouth	Categorical	**1**-Normal; **2**- Somewhat dry; **3**- Very dry
Skin turgor (slow/very slow)	Categorical	**1**- Slow/Very slow **0**-Normal
Mental Status	Categorical	**0**-Normal; **1**- Restless/Irritable; **2**-Lethargic/Unconscious
Rectal prolapse	Binary	**1**- Yes; **0**-No
Bipedal edema	Binary	**1**- Yes; **0**-No
Abnormal hair	Binary	**1**- Yes; **0**-No
Under Nutrition	Binary	**1**- Yes; **0**-No
Dehydration	Categorical	0-None; 1- Some; 2- Severe
Vesikari Score	Categorical	**1**-Mild; **2**- Moderate; **3**- Severe
ORS at facility	Binary	**1**- Yes; **0**-No
Zinc at facility	Binary	**1**- Yes; **0**-No
IV rehydration	Binary	**1**- Yes; **0**-No
any_antibiotic	Binary	**1**- Yes; **0**-No
Cipro_ceft	Binary	**1**- Yes; **0**-No
**Diagnosis**		
Malaria	Binary	**1**- Yes; **0**-No
Dysentery	Binary	**1**- Yes; **0**-No
**Anthropometric Measurements**		
Stunting	Categorical	**0**-None; **1**- Mild; **2**- Moderate; **3**- Severe
Wasting	Categorical	**0**-None; **1**- Mild; **2**- Moderate/Severe

Table S2: Hyper parameter evaluation

**Model**	**Hyper parameters**
Random Forest (RF)	Number of Trees (ntree): Evaluated values: 100, 200, 500<br>Number of Variables Tried at Each Split (mtry): Evaluated values: 2, 4, 6, 8<br>***Best Hyper parameters: ntree = 500, mtry = 6***
Support Vector Machine (SVM)	Cost (C): Evaluated values: 0.1, 1, 10, 100<br>Kernel Type: Linear Kernel<br>Gamma (γ): Evaluated values: 0.01, 0.1, 1<br>***Best Hyper parameters: C = 10, γ = 0.1***
Artificial Neural Network (ANN)	Number of Hidden Layers: Evaluated values: 1, 2, 3<br>Number of Neurons per Layer: Evaluated values: 5, 10, 20<br>Learning Rate: Evaluated values: 0.01, 0.001<br>***Best Hyper parameters: 2 hidden layers, 10 neurons per layer, learning rate = 0.001***
Gradient Boosting (GBM)	Number of Trees (n.trees): Evaluated values: 50, 100, 200<br>Interaction Depth (interaction.depth): Evaluated values: 1, 3, 5<br>Shrinkage (shrinkage): Evaluated values: 0.01, 0.1<br>***Best Hyper parameters: n.trees = 200, interaction.depth = 3, shrinkage = 0.1***
k-Nearest Neighbors (k-NN)	Number of Neighbors (k): Evaluated values: 3, 5, 7, 9<br>Distance Metric: Euclidean distance<br>***Best Hyper parameters: k = 5***

Table S3: Comparison of baseline characteristics among pediatric patients in development and temporal validation datasets.

**Characteristics**	**VIDA**	**EFGH**	**P-value**
	**n1,482**	**n=685**	
	**n (%)**	**n (%)**	
Median Age in Months [IQR]	15.0 [9.0-25.0]	13.6 [8.9-20.4]	**0.0356**
Age Category			
0-11 months	557 (37.6)	295 (43.1)	**<0.001**
12-23 months	508 (34.3)	274 (40.0)	
24-59 months	417 (28.1)	116 (16.9)	
Stool Count			
3	267 (18.0)	162 (23.7)	**0.001**
4-5	818 (55.2)	324 (47.3)	
≥ 6	397 (26.8)	199 (29.0)	
Median diarrhea days [IQR]	3 [2-4]	3 [2-3]	**0.025**
Vomiting	843 (56.9)	346 (50.5)	**0.006**
No. of vomit			
0	639 (43.1)	339 (49.5)	**0.001**
1	167 (11.3)	95 (13.9)	
2-4	541 (36.5)	208 (30.4)	
≥ 5	135 (9.1)	43 (6.2)	
Median vomit days [IQR]	1 [0-2]	1 [0-2]	**0.001**
Rotavirus vaccination	1,175 (84.6)	452 (76.5)	**<0.001**
Skin turgor (slow/very slow)	614 (41.4)	137 (20.0)	**<0.001**
Median Respiratory rate [IQR]	36.5 [31.5-41.0]	33.0 [28.0-39.0]	**<0.001**
Vesikari Score			
Mild	145 (9.8)	402 (58.7)	**<0.001**
Moderate	630 (42.5)	114 (16.6)	
Severe	707 (47.7)	169 (24.7)	
Median Vesikari score [IQR]	10 [8-13]	8 [6-10]	**<0.001**
Dehydration			
None	82 (5.5)	180 (26.3)	**<0.001**
Some	1,012 (68.3)	483 (70.5)	
Severe	388 (26.2)	22 (3.2)	

Table S4. Longer Duration Diarrhea (LDD) prediction models with no sub-sampling technique used in the resampling procedure: Model Performance

	**LDD Prediction with no sub-sampling technique used in the resampling procedure**						
**Algorithm**	**Sensitivity % [95% CI]**	**Specificity % [95% CI]**	**PPV % [95% CI]**	**NPV % [95% CI]**	**F1-Score % [95% CI]**	**AUC % [95% CI]**	**PRAUC % [95% CI]**
RF	47.9 [38.7-57.2]	84.9 [79.8-89.1]	60.0 [49.4-69.9]	77.5 [72.1-82.3]	53.3 [25.6-69.9]	80.3 [75.6-85.0]	89.0 [86.4-92.1]
GBM	52.9 [43.6-62.2]	85.7 [80.7-89.7]	63.6 [53.4-733.1]	79.3 [74.0-84.0]	57.8 [33.2-71.9]	76.6 [71.4-81.7]	86.7 [84.1-89.8]
NB	49.6 [40.3-58.9]	87.3 [82.5-91.1]	64.8 [54.1-74.6]	78.5 [73.2-83.2]	56.2 [29.7-69.6]	77.6 [72.4-82.8]	86.0 [82.9-89.2]
LR	51.3 [41.9-60.5]	90.8 [86.6-94.1]	72.6 [61.8-81.8]	79.7 [74.6-84.2]	60.1 [35.3-66.9]	81.1 [76.4-85.9]	89.0 [84.9-92.8]
SVM	48.7 [39.5-58.1]	90.0 [85.6-93.5]	69.9 [58.8-79.5]	78.8 [73.6-83.3]	57.4 [30.8-72.5]	80.5 [75.6-85.4]	88.2 [84.3-92.6]
KNN	43.7 [34.6-53.1]	88.8 [84.3-92.5]	65.0 [53.5-75.3]	76.9 [71.6-81.6]	52.3 [22.1-66.7]	75.1 [69.7-80.5]	84.7 [82.4-87.3]
ANN	52.1 (042.8-61.3]	90.4 [86.1-93.8]	72.1 [61.4-81.2]	79.9 [74.8-84.4]	60.5 [36.1-67.1]	81.2 [76.4-86.0]	89.0 [85.1-92.9]

*RF-Random Forest; GBM-Gradient Boosting; NB- Naïve Bayes; LR-Logistic Regression; SVM- Support vector machine; KNN-K-nearest neighbors; ANN-Artificial Neural Networks; 95% CI- 95% Confidence Interval; PPV- Positive Predictive Value; NPV- Negative Predictive Value; AUC- Area under the Curve; PRAUC- Precision Recall Area under the Curve
